# Adolescent Sexual and Reproductive Health Services and Implications for the Provision of Voluntary Medical Male Circumcision: Results of a Systematic Literature Review

**DOI:** 10.1371/journal.pone.0149892

**Published:** 2016-03-03

**Authors:** Michelle R. Kaufman, Marina Smelyanskaya, Lynn M. Van Lith, Elizabeth C. Mallalieu, Aliza Waxman, Karin Hatzhold, Arik V. Marcell, Susan Kasedde, Gissenge Lija, Nina Hasen, Gertrude Ncube, Julia L. Samuelson, Collen Bonnecwe, Kim Seifert-Ahanda, Emmanuel Njeuhmeli, Aaron A. R. Tobian

**Affiliations:** 1 Johns Hopkins Center for Communication Programs, Baltimore, Maryland, United States of America; 2 Population Services International (PSI), Harare, Zimbabwe; 3 Johns Hopkins University School of Medicine, Baltimore, Maryland, United States of America; 4 United Nations Children's Fund (UNICEF), New York, New York, United States of America; 5 Ministry of Health, Dar es Salaam, Tanzania; 6 Office of the U.S. Global AIDS Coordinator, U.S. Department of State, Washington, DC, United States of America; 7 Ministry of Health and Child Welfare, Harare, Zimbabwe; 8 World Health Organization (WHO), Geneva, Switzerland; 9 National Department of Health, Pretoria, South Africa; 10 United States Agency for International Development (USAID) Washington/Global Health Bureau/Office of HIV/AIDS, Washington, DC, United States of America; London School of Hygiene and Tropical Medicine, UNITED KINGDOM

## Abstract

**Background:**

Voluntary medical male circumcision (VMMC) is a critical HIV prevention tool. Since 2007, sub-Saharan African countries with the highest prevalence of HIV have been mobilizing resources to make VMMC available. While implementers initially targeted adult men, demand has been highest for boys under age 18. It is important to understand how male adolescents can best be served by quality VMMC services.

**Methods and Findings:**

A systematic literature review was performed to synthesize the evidence on best practices in adolescent health service delivery specific to males in sub-Saharan Africa. PubMed, Scopus, and JSTOR databases were searched for literature published between January 1990 and March 2014. The review revealed a general absence of health services addressing the specific needs of male adolescents, resulting in knowledge gaps that could diminish the benefits of VMMC programming for this population. Articles focused specifically on VMMC contained little information on the adolescent subgroup. The review revealed barriers to and gaps in sexual and reproductive health and VMMC service provision to adolescents, including structural factors, imposed feelings of shame, endorsement of traditional gender roles, negative interactions with providers, violations of privacy, fear of pain associated with the VMMC procedure, and a desire for elements of traditional non-medical circumcision methods to be integrated into medical procedures. Factors linked to effective adolescent-focused services included the engagement of parents and the community, an adolescent-friendly service environment, and VMMC counseling messages sufficiently understood by young males.

**Conclusions:**

VMMC presents an opportune time for early involvement of male adolescents in HIV prevention and sexual and reproductive health programming. However, more research is needed to determine how to align VMMC services with the unique needs of this population.

## Introduction

Voluntary medical male circumcision (VMMC) is a central pillar of current efforts to prevent HIV in sub-Saharan Africa [1]. Multiple clinical trials have demonstrated VMMC’s effectiveness in reducing males’ risk of acquiring HIV through heterosexual intercourse, with reductions in incidence of at least 50 to 60% [2–4]. Post-trial studies demonstrated VMMC efficacy to reduce HIV acquisition increased to above 70% over time [5]. Statistical modeling suggests scaling up VMMC services can substantially alter the trajectory of the HIV epidemic; it is projected a 30 to 50% reduction in HIV incidence can be achieved within 10 years in settings where HIV is hyperendemic, is spread primarily through heterosexual transmission, and where most men (80% or more) are not already circumcised [6]. More recent studies show reaching 80% of males ages 15 to 49 in Eastern and Southern Africa with VMMC services by 2015 could avert 3.36 million infections and 386,000 deaths by 2025, generating cost savings of $16.5 billion [7]. A decrease in HIV incidence among men will predictably have an impact on transmission among women, with estimates of an overall 37% reduction in transmission [8]. In addition, VMMC has been shown to decrease herpes simplex virus type-2, human papillomavirus, and other sexually transmitted infections (STIs) among men, and human papillomavirus, bacterial vaginosis, and trichomonaisis among female partners [9–15].

While VMMC services typically do not specifically target adolescents, a disproportionately high volume of adolescents (ages 10 to 19) make up the total population who have received VMMC in priority countries, with estimates ranging between 34 and 55% [16]. Whether this age group is adequately served with age-appropriate counseling is less understood. A review of population-based data showed the average age of sexual debut in males in priority settings is 18 years [16], while HIV infection among males tends to peak between the ages of 20 and 30 years [17]. Delivery of VMMC to male adolescents presents an opportunity to reach a large proportion of the male population prior to or in conjunction with sexual debut. It also provides an opportunity to involve young males in a lifetime of HIV prevention and sexual and reproductive health (SRH) services.

In recognition of the greater demand for VMMC among adolescents, the *WHO/UNAIDS Joint Strategic Action Framework to Accelerate the Scale-Up of VMMC for HIV Prevention (2011)* [1] includes a call for approaches to integrate and expand VMMC services for male adolescents. The framework recommends the VMMC service delivery package, including counseling, be age appropriate [1]. However, little is known about how to maximize the engagement of adolescents in quality VMMC programming when males are not socialized to regularly use health care, and SRH care settings are not designed for teens or males [18–20].

The objectives of this review were to 1) synthesize the literature on SRH and VMMC services for male adolescents in sub-Saharan Africa to determine the best age-appropriate practices that exist for this population, and 2) based on findings, make recommendations for future areas of research needed to fully understand the criteria for effective adolescent VMMC services.

## Methods

A systematic literature review [21] (see S1 PRISMA Checklist) was conducted involving an iterative process to identify terms related to adolescent sexual health services. Search terms focused on the perspectives of clients and providers in adolescent health service delivery, parental and community support for VMMC and other health services for youth, health service satisfaction for male adolescents, and delivering effective VMMC counseling to male adolescents.

Systematic literature searches were conducted in PubMed, Scopus, and JSTOR (a database providing access to regionally focused literature). All databases were searched January through March 2014. Searches were limited to studies conducted in sub-Saharan Africa (since the 14 priority countries for VMMC scale up are located in this region [1]), published in peer-reviewed journals between January 1990 and March 2014, and written in English. The 1990 start date was chosen so as to capture articles focused on adolescent SRH services and VMMC literature that pre-dated the VMMC clinical trials.

The keyword search terms were focused on four specific variables: 1) *adolescent*: adolescent*, teen*, young adult*, youth*; 2) *health services*: health service*, health care, medical care, male circumcision, VMMC; 3) *client/provider perspectives*: health personnel attitude, provider attitude, adolescent-friendly, youth-centric, patient preference, counseling; 4) *existing adolescent programming*: social marketing, intervention*, best practice*, program*, family planning, health promotion, health education, HIV infection/prevention/control, prevention. When performing database searches, an asterisk signifies multiple options for the ending of a word. Adolescent* signifies a search for “adolescent” and “adolescents.” Detailed search terms used in PubMed (as an example) are provided in Table 1.

**Table 1:** Key Search Terms for PubMed Database.

**Adolescent**	adolescent"[MeSH] OR adolescent*[tiab] OR teen*[tiab] OR young adult[MeSH] OR young adult*[tiab] OR youth*[tiab] OR homeless youth [MeSH]
**Health Services/Access to Health Services**	adolescent health services[MeSH] OR health service*[tiab] OR health care[tiab] OR medical care[tiab] OR accessibility, health services[MeSH] OR health service* accessibility[tiab] OR access to health service*[tiab] OR access to health care[tiab] OR access to medical care[tiab] OR access* health service* [tiab] OR access* health care[tiab] OR access* medical care[tiab]
**Provider and Client Perspectives/Making Services Adolescent Friendly**	attitude of health personnel[MeSH] OR provider attitude[tiab] OR adolescent-friendly[tiab] OR youth-centric[tiab] or youth centric[tiab] or adolescent friendly[tiab] or perspective*[tiab] or attitude*[tiab] OR patient preference[MeSH]
**Existing Programming for Adolescents**	social marketing[tiab] OR intervention*[tiab] OR best practice*[tiab] OR program*[tiab] OR family planning[tiab] OR prevention[tiab] OR health promotion[tiab] OR health education[tiab] OR "HIV Infections/prevention and control"[MeSH] OR "health education"[MeSH] OR "health promotion"[MeSH] OR "primary prevention"[MeSH] OR "preventive health services"[MeSH] OR counseling[tiab]OR counseling[MeSH]
**Male Circumcision**	circumcisions, male[MeSH) OR VMMC[tiab] OR male circumcision [tiab]

* Denotes any variation of the word will be included in the search. For instance, “teen*” will include teen, teens, teenager, or teenagers.

A total of 1,502 peer-reviewed articles were identified through the keyword search (see data in S1 Fig). Two independent reviewers conducted title and abstract screening to determine final inclusion in the review. A total of 703 articles were excluded due to a policy focus, topic irrelevance, and/or unrelated outcomes. Disagreements regarding inclusion were resolved through consultation between team members. A total of 263 articles were included in a full text review. Of those, findings from 79 articles were included, as the remaining 184 articles were not directly relevant to the variables of interest, data were specific to adolescent girls only, and/or there was a lack of data reported on adolescents. Of the selected studies, four constituted evidence reviews, 31 utilized quantitative methodologies, including cross-sectional and population-based surveys, 35 used qualitative methods such as focus group discussions and interviews, and nine utilized mixed method approaches. Three of the reviewed studies utilized mystery client approaches to evaluate the quality of adolescent SRH services. Fifty-one articles were dedicated to SRH service delivery, with three of these addressing issues surrounding VMMC. Twenty-eight articles focused on VMMC services with a specific mention of younger men or male adolescents.

Adolescence is generally defined as the period between 10 and 19 years of age [22]. Among the reviewed studies focused on SRH services for youth, only 14 focused specifically on populations in this age bracket; there were 27 studies where ages of participants were clearly identified that combined research on younger adolescents (10 to 15 year olds) with older youth (20 to 24 years), with one study including 25 to 26 year olds and another 25 to 30 year olds. SRH studies also included research with health service providers and articles that did not specify the age of participants, referring to them only as “teenagers” or “youth.” Out of the 29 VMMC studies, nine focused on adolescents ages 13 to 18 and young males ages 19 to 24; the remainder were investigations of men ages 15 and over or 18 and over and included mention of younger clients’ needs.

While we were most concerned with studies focused on adolescents ages 10 to 19 years old, the lack of information for this age group led to us expand the search to include young people as old as 24 years. The Society for Adolescent Medicine [23] applies the term “adolescent medicine” to health care, professional training, health research, and advocacy related to persons ages 10 to 25 years. We refer to this broad age group (10 to 24 years old) as “adolescents” or “young males” interchangeably throughout the remainder of the paper. Specific age ranges were noted where known. Articles were included in the final review if they focused on males under the age of 25 and discussed at least one of the following: 1) factors related to differences in how male and female adolescents are educated about HIV and reproductive health, 2) barriers to male adolescents seeking VMMC services, 3) an analysis of provider/educator/parent and adolescent outlook on the quality of health services specific to male adolescents, and 4) VMMC studies with stratified results for male adolescents. Articles were excluded if they provided purely biomedical or epidemiological reports on VMMC and/or did not include factors associated with improved health service delivery to male adolescents.

Experts from the World Health Organization, U.S. President’s Emergency Plan for AIDS Relief, United Nations Children’s Fund, and other implementing partners were invited to participate in an Adolescent VMMC Technical Advisory Group, including in-country Ministry of Health partners in Zimbabwe, South Africa, and Tanzania. The Advisory Group reviewed the final reference list to ensure key articles were not missed in the review. The Advisory Group, established to advise on an assessment of adolescent VMMC in-service communication, suggested two additional articles and one note of evidence. These experts also supplied seven existing guides on service delivery and the health of adolescents produced by WHO [20, 24–27], UNICEF [28], and PEPFAR [29]. These guidelines were not included in the synthesis of the peer-reviewed literature.

## Results

Table 2 briefly summarizes relevant key findings from studies included in the final review. Overall, the review revealed a lack of literature on VMMC service delivery to male adolescents in particular. Studies focused more generally on SRH services for adolescents and VMMC services for males overall. Articles included in the final review tended to cluster into three categories: 1) barriers to SRH, including VMMC services for adolescent males, 2) the role of non-medical male circumcision, and 3) facilitators for increasing SRH and VMMC service access and satisfaction.

**Table 2:** Studies of Sexual and Reproductive Health and VMMC Service Seeking Behavior and Satisfaction Among Male Adolescents in Sub-Saharan Africa.

Author, Date	Country	Sample	Study Methodology	Key Findings
Abdool Karim et al., 1992	South Africa	4 teenage mystery clients	Mystery client approach	Teenage clients experienced challenges in accessing clinics; males felt discomfort waiting in line with women and children
Ahlberg, Jylkäs, Krantz, 2001	Kenya	2267 F, 2023 M ages 11–20	Video screening followed by structured questionnaires	Myths and misconceptions among adolescents: boys understand few common SRH facts (e.g., pregnancy); boys viewed girls as STI carriers, believed girls responsible for preventing pregnancy
Ahmed et al., 2009	South Africa	15 life-orientation teachers: 3 F/12 M	Individual interviews	Educators uncomfortable discussing sex with adolescents; saw abstinence as only approach for teaching
Ajuwon et al., 2006	Nigeria	624 students; mean age 16.5, range 10–26	Cross-sectional survey in 18 public secondary schools	Fewer boys reported having romantic relationships, but reported more likely to ever had sex; teachers rated face-to-face teaching of sexual health as complex
Akpabio et al., 2009	Nigeria	339 students from 3 urban secondary schools ages 9–20	Pre/post-intervention survey assessing nurse-delivered HIV prevention, SRH education	Health education involving nurses more potent than by parents only; older students had better attitudes toward HIV prevention
Amsale, Berhane, 2012	Ethiopia	3543 adolescents ages 15–24; 49.5% M, 50.5% F; 96 students in FGDs	Cross-sectional surveys and FGDs	FGDs revealed disappointment and distrust of health providers; 65% delayed STI treatment—felt health professionals unfriendly; 70% could not obtain treatment because most institutions only open during school; 81% stated health professionals unfriendly; FGD participants reported lack of adolescent-targeted health services
Asekun-Olarinmoye et al., 2011	Nigeria	350 participants ages 10–19	Cross-sectional survey	Parents had significant influence on involvement of youth in sexual activity.
Babalola, 2006	Tanzania	1523 F, 1200 M ages 15–24	Cross-sectional quantitative study	Young men significantly more likely to have been exposed to HIV/AIDS information.
Balfour et al., 2013	South Africa	498 M/472 F, grades 5–12	Cross-sectional survey	Involvement in extra-curricular activities improved self-efficacy to prevent HIV.
Barnett, 1997	N/A	N/A	Review of best practices	Adolescent involvement in design/implementation of sexual health programming crucial to ensuring programming meets their needs.
Bastien, 2008	Tanzania	1007 youth ages 13–18	Structured interviews	More boys than girls knew condoms prevented HIV; older males in urban areas have most knowledge
Betts, Peterson, Huebner, 2003	Zimbabwe	556 M, 174 F sexually active, in school youth ages 12+	School-wide surveys	Boys engaging in safe sex more likely to report parents there when when needed, were older, spent more time in extracurricular activities; boys worried less about getting HIV compared to girls
Bosmans et al., 2006	Democratic Republic of Congo	117 adolescents ages 13–16	11 FGDs with adolescents; IDIs with program managers; 1 FGD with street youth & peer educators	Adolescent sexuality taboos impeded educators and SRH program managers from addressing issues in non-stigmatizing manner.
Bridges et al., 2012	South Africa	204 fathers, 204 mothers, 237 uncircumcised sons ages 14–30	Random sampling household survey	Most valued features in VMMC services: required follow-up visit, having lower infection rate, less pain, preference for male staff
Chandra-Mouli et al., 2013	Tanzania	N/A	Review of National Guidelines	Discussed standards for youth health in Tanzania, implementation advances in country; underlined importance of standardized services for youth to improve quality and confidentiality
Diale, Roos, 2000	South Africa	20 youth (10 M, 10 F)	Exploratory descriptive study	Community nurses stigmatized young clients; were overall unapproachable for adolescents
Downs et al., 2013	Tanzania	10 FGDs with 67 participants ages 18+	FGDs	Belief MC is 'modern practice' promoting cleanliness, prevents disease; belief women influence decision to undergo VMMC, especially mothers; some worry VMMC promotes promiscuity; parents of children in some tribes embarrassed if child circumcised; in urban areas young uncircumcised males stigmatized
Doyle et al., 2010	Tanzania	13,814 youth ages 15–30	Cross-sectional survey	In absence of community support, teacher-led, peer-assisted in-school sexual health and HIV prevention education intervention failed to impact risk behavior long term.
Erulkar et al., 2005	Kenya, Zimbabwe	1,344 youth ages 10–19 in Kenya; 539 youth in Zimbabwe	Baseline, endline surveys before/after youth activities implemented	Most important issues—Kenya: short waiting time, low cost or free, 'one-stop shop', friendly staff; Zimbabwe: confidentiality, nurse takes time, short wait time, 'one-stop shop', low cost/free; Barriers: lack of knowledge of service locations, costs
Erulkar et al., 2006	Ethiopia	1000 adolescents ages 10–19	Population-based survey	12% of adolescents visited a youth center; peer education reached 20%; centers more effectively reached older boys (nearly 1/3 of boys ages 15–19); boys more likely to have utilized both service types than girls; older youth more likely to utilize services than younger youth
Forrest et al., 2009	South Africa	2 FGDs with 11 M, 8 F ages 16–18	FGDs	Participants spoke of need to revise adolescent SRH services to be more youth-friendly where users could avoid stigma from CHWs.
Friedland et al., 2013	Zambia	915 adults ages 18+, 266 adolescents ages 13–17	10 True/False post-test questions after pre-VMMC counseling and HTC; 94 semi-structured IDIs with clients 1-week post-surgery	Fewer adolescents passed comprehension test than adults and had lower scores; difference in comprehension found between adults and adolescents, even controlling for education
Gasasira, et al., 2012	Rwanda	1098 M ages 15–59	Structured questionnaire on MC knowledge, attitudes, practices	37% of younger clients could not define VMMC; *motivators for VMMC*: HIV/STI prevention (69%), improving hygiene (49%); young men feared pain, especially those under age 19 (42%); 79% of fathers supported VMMC for son, 89% preferred son get circumcised before age 15
George et al., 2014	South Africa	143 in-school M ages 16+	FGDs	*Motivators*: hygiene, perceived increase in sexual pleasure, positive relationship with provider, facilities with better pain management, female partner preference, VMMC camps (more welcoming than health facilities); VMMC camps did not interfere with sports, other activities; *Barriers*: fear of HTC and HIV disclosure, especially if HTC on school grounds; family and community pressure
Greely et al., 2013	South Africa	Men and women ages 16 and older	15 FGDs (5 with circumcised men, 5 with women, 5 with uncircumcised men)	Men saw traditional MC key to becoming a “man”; uncircumcised men criticized, ridiculed, often excluded from community activities; concern with safety of traditional MC, long term complications, unsterilized equipment
Hatzold et al., 2014	Zimbabwe	2350 M ages 15–49; 1058 ages 15–24; 7 FGDs with ages 18–24	Population-based survey, FGDs	*VMMC motivators for younger men*: HIV prevention, social support, improved hygiene; *barriers*: fear of pain, HTC, myths and misconceptions about VMMC
Herman-Roloff et al., 2011	Kenya	121 participants ages 18–40	12 FGDs	*Barriers*: time away from work, culture and religion, possible adverse events, abstinence period, fear VMMC will make a man promiscuous; *motivators*: hygiene, social pressure, protection against HIV, improved sexual performance and satisfaction, ages 11–18 ideal time for VMMC
Hughes, McCauley, 1998	N/A	N/A	Review of best practices/evidence	Teachers, health providers lacked preparedness to discuss sexuality with adolescents.
Jayeoba et al., 2012	Botswana	269 M ages 13–18; 210 parents/ guardians	Cluster design survey	80% of boys correctly described MC; 76% of boys said MC reduces HIV risk; 75% of boys wanted VMMC after information session; 96% of parents/guardians wanted VMMC for boys; *boys’ concerns*: pain (49%), health problems (19%); *motivators*: protection from HIV (42% for boys; 40% of parents/guardians), protection from other illness (47% of parents/guardians)
Kaponda et al., 2007	Malawi	196 youth ages 10–19; parents	FGDs	Parents requested different content for 10–12, 13–15, 16+ year olds for HIV prevention; 10–12 years received no info on condoms, sexual development; emphasis on personal, general, community hygiene, HIV prevention; 13–15 years received no condom content but received info on sexual development, abstinence; 16+ years received info on condom use, sexual development
Karim et al., 2003	Ghana	3739 unmarried M/F ages 12–24	Nationally representative survey	Communication with family about avoiding sex associated with lower chance having had sex among M; friends’ opinions associated with having had sex for M; only few of those sexually experienced reported condom use during first sex—18% of M, 27% of F; reported levels of condom use at last sex were higher (43% and 37%, respectively); condoms used inconsistently: 24% of M, 20% of F reported always used condom with last or current partner
Khumalo-Sakutukwa et al., 2013	South Africa, Zimbabwe	FGDs with 23 participants in Zimbabwe, 33 in South Africa, including 16 M, 17 F ages 18–24	4 FGDs, 19 KIIs	In traditionally non-circumcised communities, younger men ashamed of being emasculated with VMMC; M were keen to learn about health benefits, how VMMC protects against HIV; appreciated improvement in hygiene, reduced pain during sex, increased sexual pleasure; females positive about VMMC and spoke about improved hygiene, increased sexual potency
Kiapi-Iwa, Hart, 2004	Uganda	Youth ages 10–21 attending school	Cross-sectional survey; IDIs with youth and providers	Youth wanted information on sexuality; valued confidentiality and rapport with providers most in regards to service quality
Kilima et al., 2012	Tanzania	601 parents; 24 traditional circumcisers; 38 health workers; 18 district/16 national stakeholders	Cross-sectional simple random sampling survey; IDIs	59% preferred traditional MC because of ceremonial aspects; disadvantages of traditional MC included pain (63.4%), high cost (50%); 52.8% preferred VMMC over traditional MC, but varied by tribe
Kim, Marangwanda, Kols, 1997	Zimbabwe	Clients ages 10–24 at 38 health clinics	418 observations of counseling sessions with youth <16 years, structured questionnaire	Youth felt rushed, unable to ask questions; providers frequently expressed judgment towards patients
Kong, 2012	Uganda	2137 VMMC trial participants, 48.5% <25 years	Prospective cohort study of uncircumcised HIV negative men at time of last visit	No significant behavioral disinhibition; among circumcised men, number in single partnership increased; among men who did not undergo VMMC, multiple partners increased; no significant differences in condom use between circumcised/uncircumcised
Kunene, 1995	South Africa	100 M, 110 F youth ages 12–19	Descriptive study using a structured questionnaire	89% of boys found youth health center beneficial; found it easier to discuss sexual issues with unknown people; *positives*: allowed to discuss feelings, make own decisions on sexual matters; *negatives*: lack of privacy, more group than individual guidance
Langhaug et al., 2003	Zimbabwe	Youth ages 16–19, Nurses	6 FGDs with youth, 4 with nurses; community meeting observation	Service delivery judgmental—lacked confidentiality and privacy; youth felt lack of privacy; providers said to break youth trust; clinics closed during out of school hours
Lanham et al., 2012	Kenya	64 F ages 18–35	20 IDIs, 4 FGDs	All women heard about partial protection from VMMC; radio, community meetings, clinics best way to reach females; most couples discussed VMMC before procedure; women encouraged procedure
Leichliter et al., 2011	South Africa	28 M ages 18–24	FGDs with young men attending health clinics	Men felt female staff did not respect their rights; felt visits and interactions were unpleasant; most men seeking STI care reported not receiving genital exams from female nurses—testing felt inadequate
Lesedi et al., 2011	Botswana	110 youth ages 15–29	Quantitative survey	26% said health providers lacked respect for youth; provider attitudes greatly impacted youth perspectives; 64% felt wait time was excessive
Lissouba et al., 2011	South Africa	1198 M ages 15–49	Cross-sectional biomedical survey: face-to-face structure questionnaire, HIV testing	Most agreed circumcised men could become HIV+, should use condoms; 81% of uncircumcised would undergo VMMC if it was free, done by doctor; most frequent reasons for not circumcising: pain (21.5%), not cultural (12.6%), risks (10%), cost (6.2%); among men with intention to have VMMC, 72.4% had VMMC through this study
Lukobo, Bailey, 2007	Zambia	M and F ages 17–81	34 FGDs—17 with M, 17 with F; two FGDs with M median age 24, FGDs with parents	Most said would take cons of MC if informed of advantages/ disadvantages, saw benefit, if MC was free; many concerned with pain and healing process; most preferred MC before puberty—believed less painful, would heal faster; non-circumcising communities preferred MC ages 7–13
Lundsby, Dræbel, Meyrowitsch, 2012	Zambia	13 recently circumcised M ages 21+	SSIs	Participants viewed VMMC positively—improved hygiene and disease prevention, enhanced sexual performance; some did VMMC along with friends and shared experiences with one another
MacPhail et al., 2009	South Africa	1736 youth ages 15–19, 2322 ages 20–24	Analysis of national youth survey	Reporting having been tested for HIV among sexually experienced young men associated with ever talking to parents about HIV/AIDS
Mark et al., 2012	South Africa	199 M ages 15–42	Interviewer-administered questionnaire, clinical examination	74% self reported MC, remaining planned to be circumcised; median age of MC was 21; 92% had MC performed by "old village man," 6% by traditional healer, 0.5% by doctor/nurse; religion most frequent reason for MC, followed by pleasing family, becoming a man; of those with sons, 16% willing to let them undergo VMMC instead of traditional MC
Marston et al., 2013	Cameroon	1754 youth ages 12–22	Longitudinal quantitative survey	Poor parent supervision is a predictor of sexual debut among males
Mashamba, Robson, 2002	Zimbabwe	30 youth ages 10–24	Exit interviews and FGDs	Cultural taboos influenced 10–14 year olds; FGD participants reluctant to discuss issues of sexuality, claimed FP is for adults
Mathews et al., 2009	South Africa	4 M, 6 F youth	Qualitative review of experiences in mystery client scenario	Breaches of privacy, confidentiality in adolescent service delivery; negative provider attitudes
Meekers, Klein, 2002	Cameroon	1284 unmarried youth < 24 years	Multi-stage stratified design with quantitative survey	Parental support associated with higher level of condom use.
Miles, 2001	Gambia	48 sexually active F, 49 M ages 15–24	12 single gender FGDs	Top reason for not seeking STI treatment was shame.
Mmari, Magnani, 2003	Zambia	200 youth ages 11–24; 60 clinic client interviews with youth ages 15–24; IDIs with nurses (30) receptionists (10), cashiers (10)	Qualitative evaluation of pilot interventions targeting improvement in adolescent-friendly services	Quality of adolescent-friendly services improved via community support.
Mngadi et al., 2008	Swaziland	58 healthcare providers delivering services to adolescents	Exploratory study using anonymous questionnaires	22% did not provide condoms to adolescents because of institutional religious principles; 36% advocated condoms be given to sexually active male adolescents; only 1 provider discussed masturbation
Mukuka, Slonim-Nevo, 2006	Zambia	515 FSW ages 15–19; 518 8th grade M ages 12–15; 520 7th grade F ages 11–14	FGDs	Male adolescents reported feeling impervious to STIs; if they were already circumcised, their understanding of VMMC’s protective qualities seemed misunderstood
Nalwadda et al., 2010	Uganda	16 FGDs with 146 youth ages 15–24	FGDs	Barriers to accessing contraception: paternalistic/judgmental health providers, limited hours, long wait time, lack of youth friendly services
Naré, Katz, Tolley, 1997	Senegal	1973 F; 936 M ages 15–24	Facility surveys, FGDs, mystery clients	Privacy and embarrassment in attending SRH services in community sites where young people felt judged.
Ndubani et al., 2003	Zambia	79 M ages 16–25	SSIs in randomly selected communities	In absence of information, young men obtained info on HIV/SRH from peers/elderly men, reinforcing risky sexual practices.
Ngalande et al., 2006	Malawi	318 M, F ages 15–80	FGDs	Hygiene important reason for wanting VMMC; younger men wanted MC to access more women; believed they could give/receive more sexual pleasure; *Barriers*: fear of infection, low confidence in safety, excessive bleeding, pain; 12 years most preferred age for MC
Niang, Boiro, 2007	Senegal; Guinea-Bissau	Not specified (younger men mentioned)	FGDs with men and women; Participant observation; KIIs	VMMC practitioners should take into account MC’s link to religion and culture.
Njue et al., 2009	Kenya	321 M ages 12–15 M; 394 F ages 14–18	FGDs with youth, IDIs with teachers	Lack of openness around sex from educators results in discomfort for students and receipt of prescriptive, inaccurate information including threats and fear messaging.
Obure et al., 2009	Kenya	126 M, 107 F out of school ages 15–34	FGDs	*Barriers*: pain, fear of loss of cultural identity, healing complications/time, cost, stigma, fear of inability to sexually please women; more counseling needed—should emphasize hygiene, other benefits instead of HIV; *motivators*: hygiene, reduced risk of STI/HIV, easier condom use, cultural integration (ability to be with women from tribes that circumcise), increased sexual satisfaction
Okonofua et al., 1999	Nigeria	48 providers serving adolescents	48 IDIs with traditional and formal health practitioners; site visits	Formal health workers failed to discuss STIs, condom use with adolescents due to religious doctrine.
Pattman et al., 2003	Botswana, Kenya, Rwanda, South Africa, Tanzania, Zambia, Zimbabwe	Children ages 6–16	Same and mixed gender group interviews, video interviews	Children 6+ years old and adolescents extremely interested in topic of sexuality, teachers not ready to discuss with them; lack of knowledge and persistent interest produced many misconceptions, fed into traditional gender role stereotypes, created unhealthy gender-power dynamics
Plotkin et al., 2013	Tanzania	142 participants: 68 F, 34 M ages 18–29, 30 M ages 30+	FGDs	Young men concerned with appearance and abstinence period; knowledge of VMMC fairly high; *motivators*: peer pressure, women’s preferences, disease prevention, cleanliness; belief best to perform MC before puberty
Ragnarsson et al., 2008	South Africa	72 students ages 12–24	FGDs	Many adolescent men felt boys were like women if circumcised at a clinic; MC seen as right of passage into sex, more partners
Renju et al., 2010	Tanzania	Health workers, youth mystery clients	Questionnaires; FGDs	Mystery client experiences revealed lack of privacy and difficulty for adolescents to approach health staff.
Rijsdijk et al., 2012	Uganda	1978 youth ages 12–20; 885 M, 1093 F	School-based quantitative survey	Perceived social norms and attitudes towards condom use significantly associated with delayed intercourse and condom use.
Schatz, Dzvimbo, 2001	Zimbabwe	3429 students ages 15–19; 49% M	Structured survey	Traditional healers often sought by youth because are more tolerant of sexuality.
Schenk, et al., 2012	Zambia	36 parent/ guardian FGDs; comprehension test: 159 adults, 69 adolescents; SSI: 28 adolescent M ages 13–17; KII: 2 F, 11 M	SSIs with MC clients 1 week post-surgery; parent/guardian FGDs (3 circumcised sons, 3 non); 13 KIIs with providers, community reps, other stakeholders; comprehension assessment	Adolescents less likely than adults to report comfort with MC decision (44% vs. 13%); adolescents more likely to make final decision (89% adolescents, 69% adults); comprehension high among adolescents and adults; 75% of consent forms signed by parent, 13% by guardian, 12% by older sibling; some felt minors should be able to undergo MC without parental consent
Ssekubugu et al., 2013	Uganda	M ages 15–19, 20–35, 36–49	33 IDIs, 23 FGDs	*Barriers*: fear of pain, medical complications, belief VMMC leads to infertility; some decliners did not believe efficacy of HIV prevention as they knew circumcised men who died; *motivators*: prevention of STIs, hygiene, peer influence; HTC and partner influence were barriers and motivators for different individuals
Tesso et al., 2012	Ethiopia	2269 youth ages 10–24; 54.5% ages 15–19	Community-based cross-sectional household survey; 13 FGDs	*Barriers in discussing SRH with parents*: fear of embarrassment, sexual taboos, parent failure to listen, parent lack of interest to discuss
Wambura et al., 2011	Tanzania	170 M; 189 F ages 18–44	Cross-sectional questionnaire	97% M, 95% F supported VMMC for their sons; 73% VMMC preferred before age 12—faster wound healing, bleeding/pain believed to be less when young
Warenieus et al., 2006	Kenya, Zambia	Kenya: 322 midwives: Zambia: 385 who deliver services to youth	Cross-sectional survey	Majority of midwives in Kenya and Zambia expressed judgmental opinions of adolescent sexual behavior.
Warenius et al., 2007	Zambia	716 students ages 11–22; 354 F, 362 M	Questionnaires	Poor knowledge of SRH among students; curiosity about MC and protection against HIV; have many questions for parents, health providers—avoid questions by youth inquiring about SRH and sexuality in general
Wilcken et al., 2010	Uganda	267 adults ages 25+, 185 youth ages 14–24	Cross-sectional survey	76.5% of young people aware of VMMC as means of HIV prevention; media listed as main source of VMMC information followed by family/friends, teachers; *reasons for MC*: religion, improved hygiene, culture; 13% listed HIV prevention as a motivator
Wild et al., 2004	South Africa	939 students ages 12–26, 519 F	Quantitative survey in public school	Low family self-esteem associated with risky sexual behavior.
Wilson, Lavelle, Hood, 1990	Zimbabwe	156 M, 33 F, 7 undisclosed M or F; mean age 16.9	Quantitative questionnaire	Consultations and beliefs of parents in regards to condom use positively correlated with intended condom use.
Wouhabe, 2007	Ethiopia	890 M, 3988 F ages 15–24	Ethiopia Demographic Health Survey	Male youth had more SRH knowledge than females; overall awareness among both genders low
Zuurmond et al., 2012	N/A	17 studies on effectiveness of youth centers in increasing SRH service access	Systematic review	Proximity and community support of centers major factors in utilization; in 4 studies, satisfaction with centers low due to lack of privacy

CHW: Community Health Worker. F: Female. FGD: Focus Group Discussion. FP: Family Planning. FSW: Female Sex Worker. HTC: HIV Testing and Counseling. IDI: In-Depth Interview. KII: Key Informant Interview. M: Male. MC: Male Circumcision. SRH: Sexual/Reproductive Health. SSI: Semi-Structured Interview. STI: Sexually Transmitted Infection. VMMC: Voluntary Medical Male Circumcision.

### Barriers to Sexual Health Service Access and Satisfaction for Adolescent Males

Forty-six articles reported barriers to accessing health services for male adolescents, including structural factors, privacy violations, shame, limitations in sexual education for male adolescents, negative interactions with providers, and pain associated with VMMC. While the majority of these articles (37 in total) focused on barriers to SRH service delivery, there are clear implications as to how these broader themes can impact VMMC service uptake as well.

#### Structural factors

Structural barriers to SRH service-seeking highlighted by male adolescents included clinics’ limited operating hours coinciding with school and work hours and an unwelcoming atmosphere for adolescent clients [30–33]. In one study in Botswana, afternoon and evening hours were recommended by male and female adolescent clients [31]. For VMMC services in particular, studies from Kenya and Tanzania reported that taking time off from work and traveling far distances is a barrier for employed young men (ages 18 to 24) [34–36]. Another study in South Africa found that male adolescents are also less likely to respond to VMMC recruitment when engaged in sports and during school exam periods [37]. Teenage male SRH clients in another South African study reported discomfort in waiting in lines with women and young children [19]. In other studies conducted in South Africa and Zambia, male adolescents wanted to attend clinics only while being accompanied by a peer educator or to interact with and ask health-related questions only of people of a similar age [18, 19, 38].

#### Disregard for privacy

Across studies, respect for privacy and confidentiality, especially in close-knit rural communities, was reported to be a cornerstone of acceptable health service delivery for adolescents [19, 39–46]. Multiple studies highlighted adolescents’ discomfort with physical privacy constraints in health centers, such as open doors or counselors’ desks being located near a window or partition that insufficiently muted voices, but also reported being wary of potential interactions with seemingly judgmental health workers prone to gossip in communities where everyone knows each other [19, 40–42, 47, 48]. A systematic review of 17 studies addressing the effectiveness of youth centers—facilities specifically created to accommodate the needs of adolescents—in increasing SRH service uptake found these centers were rated poorly by youth when they lacked privacy [49]. In another VMMC-specific study in South Africa, fathers, mothers, and sons all valued private waiting rooms when seeking service delivery [50].

#### Shame

Despite evidence that adolescents in sub-Saharan Africa are eager to discuss sex and sexuality [51] and appreciate directness and clarity when these topics are introduced by adults [52], shame and embarrassment were characteristics of adolescents’ experiences of SRH services in the region [32, 43, 53]. These feelings were reported to be magnified by adult behavior [51]. For instance, school counselors, teachers, parents, and health staff often report being embarrassed and/or perceived as judgmental when introducing topics on sex, sexuality, and contraception [18, 46, 51, 52, 54]. Refusal on the part of teachers and health workers to discuss these topics with youth is sometimes attributed to religious doctrine [55, 56] and the belief that introducing sexual education topics and contraception to youth “increases their immorality” [19, 32, 40, 41, 43, 44, 57, 58]. This lack of comfort with discussions of sexuality and health transfers to feelings of shame and embarrassment by the adolescents as well.

A study in the Gambia found that shame was a key reason young people did not access health services, even if they had STI symptoms [53]. Other studies from Kenya, Senegal, South Africa, Uganda, and Zimbabwe reported adolescents had a fear of encountering acquaintances and general embarrassment when navigating large community clinics and hospitals to obtain services, as well as concerns that health workers were too busy in these environments to sufficiently respond to their questions [19, 32, 39, 43].

#### Limitations in sexual health education for male adolescents

As a result of teacher and health educator discomfort, male adolescents reported often receiving information that reinforces gender stereotypes and perpetuates gender inequality, such as the need for boys to be good sexual performers, or for stigmatization of sexually active girls [51, 55]. In the absence of effective education, consultations with peers on facts and advice about sexual health was found to increase responsible sexual health decision-making in a study in Tanzania [59], but resulted in receipt of erroneous information in a study in Zimbabwe [30] and reinforcement of risky sexual practices by male adolescents in a study in Zambia [60].

Some studies showed male adolescents generally lack awareness and lag behind female adolescents in knowledge about reproductive health and risks, including STIs and HIV [60–64]. Other studies showed male adolescents are generally more exposed to information about HIV/AIDS [59, 65], and data from a 2013 UNICEF report demonstrated male adolescents in sub-Saharan Africa possess better comprehensive knowledge of HIV than do female adolescents [28].

#### Negative experiences with providers

Across studies, health providers’ disregard for privacy [19, 39–44, 48], their expressions of negative judgment of adolescents seeking services [19, 32, 40, 41, 43, 44, 48, 57, 58], and general “lack of respect” for or desire to be approached by youth [33, 66] indicate some health care workers in sub-Saharan Africa have attitudes and beliefs impeding their ability to meet the needs of adolescent clients. Furthermore, adolescent patients in a study in Uganda reported feeling rushed by providers during service [41]. In another study, male clients attending community clinics in South Africa felt disrespected and “chastised” by predominantly female staff [67]. Comfort with staff [40] and staff friendliness [39] were reported by adolescents in Uganda, Kenya, and Zimbabwe to be indicative of high quality services, but these characteristics were rarely identified as sufficient.

Despite these challenges, other studies in Zimbabwe, Kenya, and Nigeria showed adolescent patients value health providers’ knowledge and opinions on matters of SRH [42], feel doctors and nurses tend to be less judgmental than teachers [55], and are receptive to HIV education involving nurses [68]. If the interaction with the providers were more respectful, this barrier to attracting youth to SRH services might be reduced.

#### Fear of pain and HIV testing in VMMC

Fear of pain was reported as a significant barrier to performing VMMC among male adolescents in Botswana, Rwanda, South Africa, and Uganda [50, 69–71]. Male adolescents in Tanzania and South Africa were also concerned with the pain resulting from the breakage of stitches if they have an erection during the healing phase [35, 37]. In the same South African study, better pain management was shown to be a facilitator for VMMC among male adolescents when compared to traditional non-medical circumcision [37]. Studies from South Africa and Zimbabwe indicated HIV testing also created a barrier to VMMC for male adolescents who feared a positive result and subsequent stigma [37, 50, 72], especially when conducted during in-school recruitment, where male adolescents thought their test results would be immediately known to everyone [37].

### The Role of Traditional Non-Medical Circumcision

Twelve articles discussed the role of non-medical circumcisers in relation to VMMC [53, 56, 62, 64, 73–80]. Three general adolescent SRH articles noted that in the absence of health workers, youth in the Gambia, Nigeria, and Zimbabwe sometimes preferred going to traditional healers for services, as such healers are more open to the idea of discussing sex, contraceptives, and reproductive health [53, 56, 73].

Studies in Senegal, Guinea-Bissau, and South Africa reported traditional non-medical circumcision in most settings includes educating boys on aspects of male strength and how to be a role model, survivor, and provider [74, 75]. Neglecting this component in VMMC and minimizing the perception of male circumcision as a right of passage into manhood can create dissatisfaction among parents and community leaders [74–76]. In one study in South Africa, male adolescents expressed concern that VMMC lacks traditional components, and youth who get circumcised in a clinical setting are “men being like women” [75]. In a study in Zambia, where male adolescents are not traditionally circumcised, there was a lot of curiosity about the procedure, and the belief that male circumcision combined with the process of initiation provided protection against HIV [62]. Since VMMC is not a widespread practice in Zambia, there was great curiosity about the relationship between the concept of 'initiation' and the risk of contracting HIV or becoming pregnant [62].

In another study conducted in a South African community where men are traditionally circumcised, 82% of fathers expressed unwillingness to allow their sons to undergo VMMC for religious and/or cultural reasons, notions of manhood, and social disapproval. Even those fathers who were more tolerant of VMMC indicated male adolescents should undergo the procedure between ages 18 to 20 [77].

Studies from Malawi, South Africa, Tanzania, and Zambia, however, found both younger men and parents prefer the procedure be performed by a health professional rather than a traditional non-medical circumciser, as they fear greater pain and an unsanitary environment associated with the latter [76, 78–81]. One study in Tanzania indicated traditional non-medical circumcisers and health professionals displayed readiness to work together to incorporate aspects of traditional education into VMMC in an attempt to attract more male adolescents to the service [76].

### Factors Increasing Sexual Health Services Access and Satisfaction

Across studies, several factors were reported to increase service uptake and satisfaction among adolescent males, including parental and community involvement, a youth-friendly service environment, other perceived VMMC benefits aside from HIV risk reduction, and proper comprehension of VMMC messages [18, 32, 37–39, 69, 70, 72, 81–83]. There were 21 articles addressing general SRH service satisfaction, and 26 addressed issues related to VMMC service satisfaction in particular.

#### Parental involvement and shared decision-making

Parental involvement in adolescent well-being was shown to impact adolescents’ health seeking behavior and other positive health outcomes in Ghana, Nigeria, and Zimbabwe [84–86]. Studies demonstrated multiple benefits of parental involvement, including improved understanding of topics related to sexual education and HIV [86, 87], increased intention to use condoms [88], increased actual condom use [89], and increase in HIV testing among young men [87]. While both male and female adolescents were interested in discussing SRH topics with their parents in a study in Ethiopia, younger males (10 to 14 years) reported the lowest levels of parental communication compared to all other age and gender groups [90]. Lack of parental supervision and involvement reported in studies in Kenya and South Africa were associated with early sexual debut, particularly among adolescent males [91], and risky sexual behavior [92].

With respect to source of VMMC awareness and receptivity, a study in Zambia reported parents were the first source of information for 61% of adolescent participants [83]. Also in Zambia, nearly all male and female focus group participants from non-circumcising districts reported they would take their sons to be circumcised at a health facility if they were provided information on the procedure and its advantages [80]. In the same study, parents also said the procedure should be free or low cost, indicating cost is sometimes a barrier to VMMC for parents [80]. In Kenya, women were the key encouraging force behind VMMC for both their partners and sons [93]. In Tanzania, fathers who themselves were not circumcised said they would support their sons doing so because they thought men seeking VMMC are a positive influence on the household [35]. Two other studies in Tanzania in areas where traditional non-medical circumcision was the norm showed more than half of surveyed parents in one study and more than 95% in the other preferred VMMC compared to traditional non-medical forms [76, 94]. This preference was attributed to the fact that VMMC was part of school initiation and the time when boys become “warriors” for the community [76, 94].

The extent of an adolescent’s involvement in decision-making about VMMC is a factor that varied by study. In Botswana, it was a shared decision between parents and male adolescents in a majority of households [70]. In another study in Zambia, two thirds of adolescents reported making a decision about VMMC and then obtaining approval from their parents [95], however, in a study in Senegal and Guinea-Bissau, adolescents’ assent was only sought by half of the participants [76]. In a traditional circumcision setting in South Africa, only 3% of the fathers stated that undergoing VMMC (as opposed to traditional non-medical circumcision) would be their sons’ decision [77]. A study in Botswana also showed the majority of adolescent males made the final decision regarding VMMC just prior to entering the clinic [70], signifying how important adolescents’ participation in decision-making could be for their perception of the procedure.

#### Community involvement

The impact of community support on VMMC uptake among adolescents has not been extensively studied, as only one study in Zimbabwe addressed this topic. This study showed that males of all ages reporting social support for VMMC from friends and peers had three times greater odds of being circumcised than those who did not receive such support [72]. Community support and acceptability of SRH interventions has been shown to influence health service uptake and improve risk prevention behaviors among adolescents [18, 96–99]. A study in Zambia evaluated the effect of three youth-friendly community- and clinic-level interventions and found community support was the only key factor in increasing health-seeking behavior among youth [18]. In the absence of community support, one study in Ethiopia showed only 12% of adolescent respondents for a population-based survey visited a youth center [97], and in another study in Tanzania, a youth-focused intervention failed to improve HIV and STI prevention behavior [96]. In Malawi, thorough consultations with adolescent and adult community representatives allowed for successful implementation of an HIV-prevention intervention for youth [98].

#### Youth-friendly services

Along with identifying barriers to adolescent service satisfaction, several studies in Kenya, Uganda, and Zimbabwe documented what adolescent clients want from health services. Friendliness and patience from medical staff and feeling relaxed and comfortable with a health care provider were named as key preferences [39–41]. Short waiting times, “one-stop-shop” approaches (where adolescents can receive other services in addition to health), and adolescent involvement in program design are highlighted in the literature as examples of adolescent-friendly best practices [39, 100].

#### Other Perceived Benefits of VMMC

A number of studies have shown male adolescents view improved hygiene as a key benefit of VMMC and associate the procedure with being modern and increased ability to sexually satisfy partners [35, 69, 72, 80, 81, 101–103]. The interest in sexual performance can be both an encouraging factor for increasing rates of VMMC and a factor in risk compensation. In Malawi, young men in particular noted during focus group discussions a desire to have access to more women as their main motivation to seeking VMMC [81]. Two studies found that adolescents and younger males were more prone to risk-taking after VMMC—in one qualitative study from Zambia, male adolescents aged 15 and younger reported they were more likely to be sexually active if they were circumcised because they believed they were impervious to STIs [104], and in another study in Zimbabwe, 22.6% thought they would not need to use condoms after VMMC [72]. Another study in Uganda, however, found risk compensation was not present among men (including adolescents) who underwent VMMC [105]. In Kenya, young men associated VMMC with potential reduction in penis size and sensitivity, thus felt the procedure would make sex less satisfying for themselves and their partners [34].

#### Understanding VMMC messages

VMMC messages properly targeted toward adolescents are key for successful service uptake and satisfaction, and eleven articles addressed this topic [37, 50, 62, 71, 72, 75, 83, 95, 101, 104, 106]. A study in Zimbabwe and South Africa found that lack of information and misconceptions about benefits of VMMC (including impact of the intervention on reducing HIV and STIs for both men and women) were the key barriers to seeking VMMC among both adults and adolescents [101]. Another study reported adolescents in Zambia and Swaziland were confused about the protective value of VMMC on HIV and STI transmission, as well as the reduction in their partners’ cervical cancer risk [83]. Furthermore, a study in Uganda showed wrongly interpreting and inflating beliefs about VMMC’s protective value reduced male adolescents’ interest in VMMC if they knew someone who was circumcised and still died of AIDS [71].

To increase understanding of VMMC, national campaigns and the media have shown effectiveness in reaching male adolescents with specific knowledge about the procedure and its protective effects [106]. In a study from South Africa, male adolescents reported valuing the information they received from interpersonal interactions with mobilizers and health providers [37]. Two studies from Uganda and Zimbabwe mentioned that participants of all ages reported primarily receiving information about VMMC from the media [72, 106].

One study focused on pre- and post-VMMC counseling procedures in Zambia and Swaziland reported these sessions have diminished effectiveness for adolescents when compared with adult men [83]. Young clients in the study scored significantly lower than adult men on a post-educational session test assessing their comprehension of the VMMC procedure, protective factors associated with it, and risk factors associated with HIV and STIs in circumcised men [83]. In another study from Zambia, adolescent VMMC clients were less likely to identify risk factors associated with VMMC surgery and more likely than adults to think that all circumcised men were HIV negative [95]. Descriptive concepts—for example, the foreskin’s ability to trap bacteria—were highlighted in studies conducted in South Africa, Swaziland, and Zambia as something well understood by adolescents [75, 83], but more complex concepts, such as risk reduction, are generally interpreted as being a guarantee against future HIV infection, as found in studies in two studies in Zambia and one in South Africa [62, 75, 104]. In another study from South Africa, male adolescents most valued counseling on specific risks and benefits of VMMC [50].

## Discussion

The literature on adolescent boys’ experiences with sexual health programming generally and VMMC specifically is limited. Given VMMC is a relatively new intervention, this dearth of published research is not surprising; nevertheless, it signals a challenge for public health in trying to reach the largest generation of males approaching adolescence in African history. Understanding how best to provide VMMC specifically and reproductive health care more generally will be essential in ensuring this generation of men is healthy and productive. Existing literature describes barriers to both accessing and appropriate delivery of services, including structural factors, imposed feelings of shame, negative interactions with providers and violations of privacy, fear of pain associated with the VMMC procedure, and a desire for elements of traditional non-medical methods of circumcision to be integrated into medical forms. Factors identified as facilitating effective youth-focused services included the involvement of parents and the community, a youth-friendly service environment, promoting additional perceived benefits of VMMC, and messages better understood by young males. These findings are also supported by global guidance documents [20, 24–26, 28, 29]. Furthermore, guidance from UNICEF, WHO, and the United Nations Population Fund promotes age-appropriate ways of introducing SRH concepts to male adolescents during VMMC counseling as a way to build lasting demand for health services among males [27].

### Recommendations for Future Research and Programming

While VMMC service delivery for adolescents is still new, clearly much more research is needed to determine how best to reach this population in order to make relevant recommendations that will have the greatest impact on HIV incidence reduction. Future research needs to explore how to appropriately tailor counseling during the VMMC process to adolescents of different ages, levels of maturity, and sexual experience. For instance, counseling appropriate for a 10 year old without sexual experience may be quite different than what is appropriate for an 18 or 19 year old with some sexual experience. Little is also known about how counselors and service providers discuss 1) sex in general, 2) the VMMC procedure and its benefits, 3) how VMMC links to sexual health services, 4) how to maintain safer sex behavior in the future when a male adolescent may not have reached sexual debut, 5) gender roles and masculinity, and 6) HIV testing and counseling and linkages to care, if needed. There is also a lack of knowledge about providers’ capacity for counseling male adolescents generally and possible ways to engage parents in prevention and messaging around HIV and VMMC. In addition, it is unknown how parents would react to counseling about prevention of STIs when their male adolescent has not reached sexual debut. These issues may also vary by setting, ethnic background, or cultural context.

Cultural, social, and gender norms; parent, partner, and community involvement; and individual factors clearly all play a role in whether or not a male adolescent is able to easily access services, receive quality care, and is satisfied with the procedure overall. It is clear from the literature that parental involvement and community support play an important role in health service access for young males. Capitalizing on these factors of influence could attract more young males to both SRH and VMMC services as communities and their leaders provide an enabling environment in which males do not feel shame in seeking services. At the same time, ensuring services for adolescent males are provided in a way that maintains their privacy, even from their parents, is also an important consideration.

Equally essential is the need for counseling and education tools designed for and tested with male adolescents. Materials that are sufficiently understood by this population, especially when focused on complex topics such as sexual risk reduction, could help to increase the effectiveness of the services. Furthermore, in countries where traditional circumcision is prevalent, programs may also consider working with traditional circumcisers to deliver VMMC as part of larger traditional ceremonies or incorporating elements of those initiation events into VMMC.

Understanding cultural differences and/or similarities for various African male adolescent populations is also key to improving the success of programs. For instance, program guidelines that inform acceptable services for male adolescents in rural Uganda may look quite different from what is acceptable for urban South African males. While international guidelines on youth-friendly services are available [107], such guidelines must be carefully adapted using formative research to identify what is appropriate and effective within each context. Program implementers can rapidly conduct this research as part of program development. Where standards based on global guidance for implementing adolescent-friendly SRH services are implemented, they have shown some impactful results if grounded in wider public health work at the national and community level [45]. Thus, integration of age and culturally appropriate VMMC programming and other SRH services for adolescents are necessary.

### Study Limitations

This review focused on studies highlighting improvements needed in SRH and VMMC service delivery to male adolescents. Distinct conclusions are difficult to draw given the evidence for adolescent populations is still thin. Furthermore, variation in study methodologies, as well as differences in the ages of adolescent participants, outcome variables, and scopes of analyses makes it difficult to fully compare studies. However, this systematic review clearly illustrates where more work is needed.

We did not assess for bias in the included studies, as we were not looking at effect sizes or impact on outcomes, but rather a combination of descriptive and (quasi) experimental studies. We also did not review the unpublished literature, and therefore might have missed key programs and studies in these areas of interest, as many such efforts conducted in the context of development are not moved to the peer-reviewed publication stage.

### Conclusion

VMMC is a highly effective intervention for curbing the HIV epidemic in sub-Saharan Africa [2–4, 7]. However, in order to seize this opportunity and effectively reach male adolescents with a meaningful, comprehensive VMMC package, more efforts are needed to effectively tailor guidelines, services, and messages to these younger clients. A more in-depth analysis of young males’ needs that takes into account their age, developmental stage, and cultural differences will help to further strengthen adolescent VMMC service delivery.

## Supporting Information

S1 PRISMA Checklist PRISMA 2009 Checklist. (DOC)

S1 Fig PRISMA flow Diagram. (PDF)
