# Supplementary figures and images for: Adolescent Sexual and Reproductive Health Services and Implications for the Provision of Voluntary Medical Male Circumcision: Results of a Systematic Literature Review
Source: PLoS One. 2016 Mar 3;11(3):e0149892. doi: 10.1371/journal.pone.0149892 (PMC4777442; doi:10.1371/journal.pone.0149892)

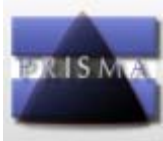

## PRISMA 2009 Flow Diagram

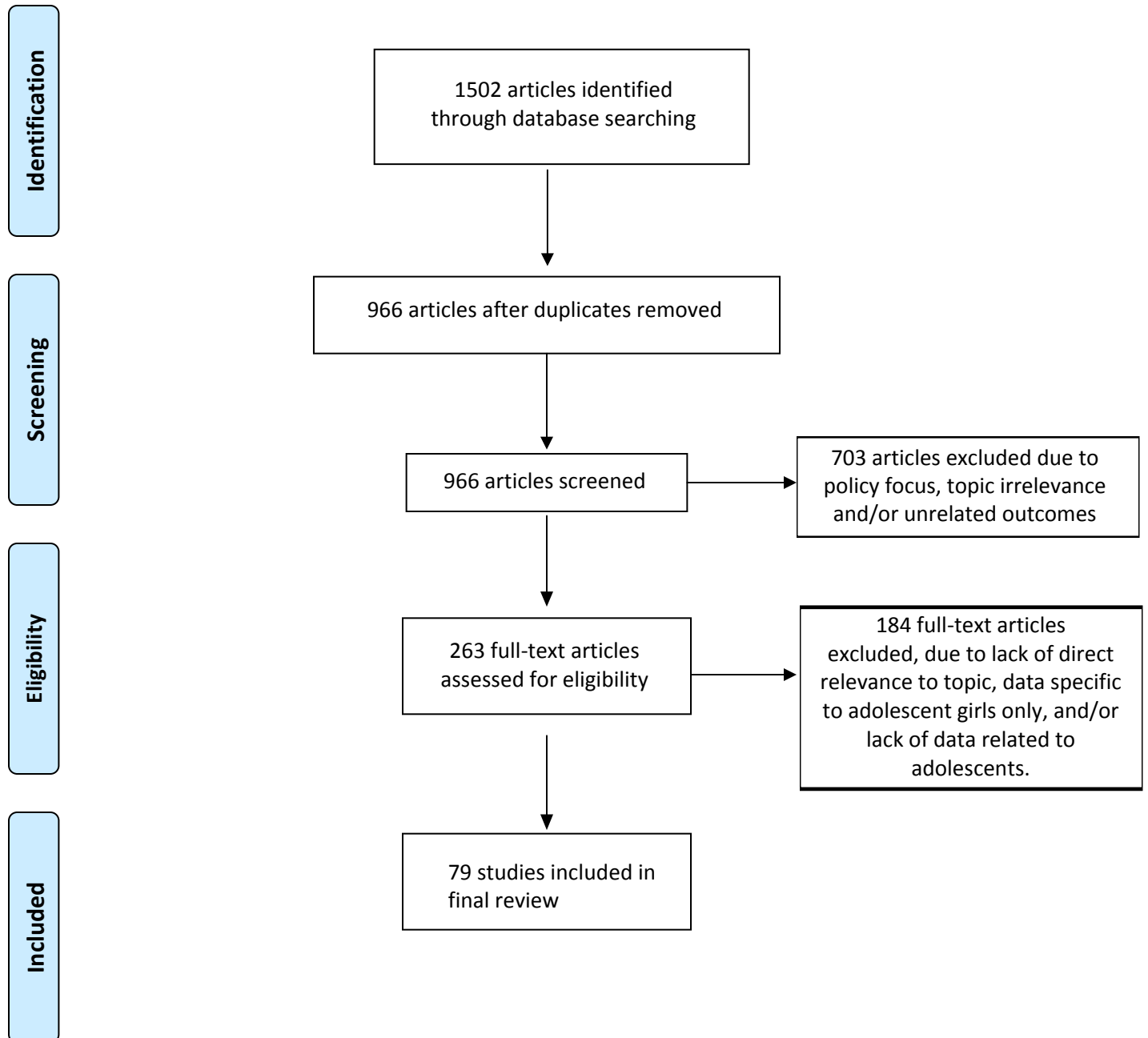

Supplement: S1 Fig — (PDF) [file pone.0149892.s002.pdf]
